# Genistein Targets STING-Driven Antiviral Responses

**DOI:** 10.1128/mbio.02064-22

**Published:** 2022-08-04

**Authors:** Tomalika R. Ullah, Katherine R. Balka, Rebecca L. Ambrose, Geneviève Pépin, Matthew C. J. Wilce, Jacqueline A. Wilce, Belinda J. Thomas, Dominic De Nardo, Bryan R. G. Williams, Michael P. Gantier

**Affiliations:** a Centre for Innate Immunity and Infectious Diseases, Hudson Institute of Medical Research, Clayton, Victoria, Australia; b Department of Molecular and Translational Science, Monash University, Clayton, Victoria, Australia; c Department of Anatomy and Developmental Biology, Monash Biomedicine Discovery Institute, Monash University, Clayton, Victoria, Australia; d Department of Biochemistry and Molecular Biology, Monash Biomedicine Discovery Institute, Monash University, Clayton, Victoria, Australia; e Monash Lung and Sleep, Monash Medical Centre, Clayton, Victoria, Australia; f Centre for Cancer Research, Hudson Institute of Medical Research, Clayton, Victoria, Australia; Johns Hopkins Bloomberg School of Public Health

**Keywords:** Genistein, STING inhibitor, cGAMP, gap junction

## Abstract

Cytoplasmic detection of DNA by cyclic GMP-AMP (cGAMP) synthase (cGAS) is an essential component of antiviral responses. Upon synthesis, cGAMP binds to the stimulator of interferon (IFN) genes (STING) in infected and adjacent cells through intercellular transfer by connexins forming gap-junctions, eliciting a strong IFN-β-driven antiviral response. We demonstrate here that Genistein, a flavonoid compound naturally occurring in soy-based foods, inhibits cGAS-STING antiviral signaling at two levels. First, Genistein pretreatment of cGAMP-producing cells inhibited gap-junction intercellular communication, resulting in reduced STING responses in adjacent cells. In addition, Genistein directly blocked STING activation by the murine agonist DMXAA, by decreasing the interaction of STING with TBK1 and IKKε. As a result, Genistein attenuated STING signaling in human and mouse cells, dampening antiviral activity against Semliki Forest Virus infection. Collectively, our findings identify a previously unrecognized proviral activity of Genistein mediated via its inhibitory effects at two levels of cGAS-STING signaling.

## OBSERVATION

Following synthesis by cGAS, cGAMP binding to STING leads to its oligomerisation and interaction with TBK1 and IKKε kinases (1–3). This results in the phosphorylation of STING and IRF3, promoting a strong antiviral response through IFN-β production (2–4). In addition to engaging with STING in infected cells, cGAMP rapidly propagates antiviral responses to uninfected cells through direct extracellular secretion (5), via gap junction intercellular communication (GJIC) (6, 7) or incorporation in viral particles (8).

**FIG 1:**
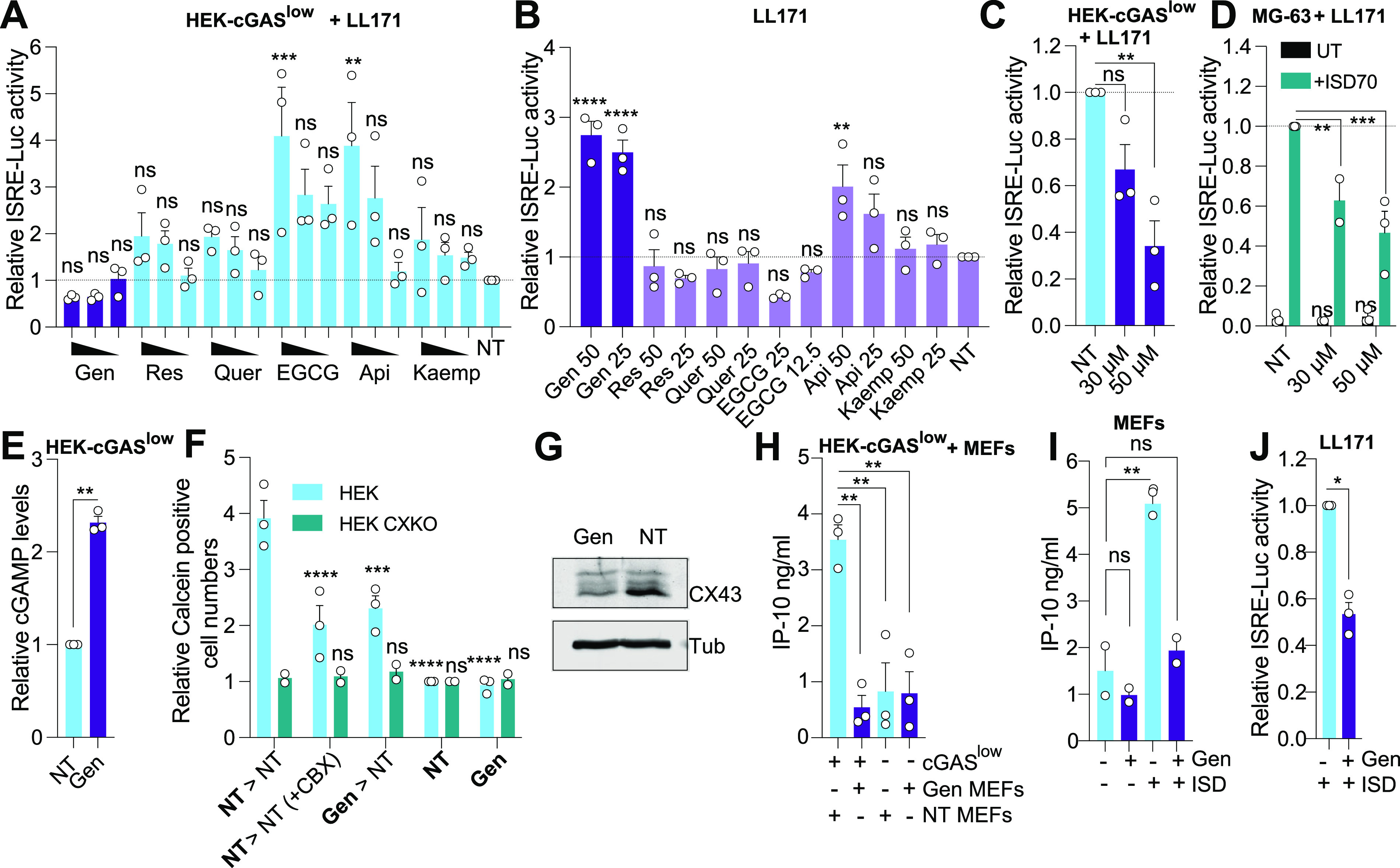
Genistein inhibits transactivation of neighboring cells. (A) HEK-cGAS^low^ cells pretreated with indicated compounds (Genistein [Gen], Resveratrol [Res], Quercetin [Quer], Apigenin [Api], and Kaempferol [Kaemp] at 50, 25, and 12.5 μM; EGCG at 25, 12.5, and 6.25 μM) for 48 h were cocultured overnight with LL171 cells. ISRE-Luciferase levels were measured from the cell lysates the next day. The data are shown relative to nontreated (NT) LL171 cells (*n* = 3). (B) LL171 cells were treated with indicated compounds for 48 h and ISRE-luciferase levels were analyzed. Data are shown relative to NT LL171 cells (*n* = 3). (C) HEK-cGAS^low^ cells pretreated with 50 or 30 μM Genistein for 48 h were collected and counted. ~20,000 HEK-cGAS^low^ cells were subsequently cocultured overnight with LL171 cells. ISRE-Luciferase levels are shown relative to NT LL171 cells (*n* = 3). (D) MG-63 cells pretreated for 48 h with 30 or 50 μM Genistein were transfected with 2.5 μg/mL of ISD70 for 4 h (after wash to remove ISD70 [7]) or untransfected (UT), and cocultured overnight with the same amount of recipient LL171 cells. ISRE-Luciferase levels are shown relative to the ISD70-treated cells (*n* ≥ 2). (E) HEK-cGAS^low^ cells were treated for 48 h with 50 μM Genistein prior to lysis and cGAMP-specific ELISA. Data are shown relative to NT HEK-cGAS^low^ cells (*n* = 3). (F) HEK-STING (HEK) and HEK-STING CX KO cells (HEK CXKO) were pretreated with 50 μM Genistein or not (NT) for ~40 h followed by treatment with 2 μg/mL Calcein-AM solution for 1 h. The donor cells (in Bold) were washed, counted, and 7,000 donor cells were cocultured (indicated with “>” sign) with ~40,000 recipient confluent cells of the same genotype, in the presence or not of CBX (100 μM). Calcein dye transfer was quantified after 4 h by counting the number of Calcein positive cells per fields. Data are shown reported to NT condition [Calcein positive cells only] (*n* = 2 for HEK CXKO and *n* = 3 for HEK). (G) Western blot analysis of CX43 levels from the lysates of MEFs treated with 50 μM Genistein for 48 h (representative of *n* = 2). (H) MEFs pretreated with or without 50 μM Genistein for ~40 h were cocultured overnight with HEK-cGAS^low^ (cGAS^low^) cells. Supernatants were collected, and mouse IP-10 levels measured by specific ELISA (*n* = 3). (I) MEFs were pretreated for 48 h with 50 μM Genistein prior to the overnight transfection with 1 μg/mL ISD45 and mouse IP-10 levels measurement by specific ELISA (*n* = 2). (J) LL171 cells were pretreated for 1 h with 40 μM Genistein, prior to transfection with 2.5 μg/mL ISD45 for 6 h. ISRE-Luciferase levels are shown relative to the ISD-treated cells (*n* = 3). (A, B, C, D, E, F, H, I, J) Each point represents the mean data for each independent experiment. Unpaired t-tests (E, J), two-way (D, F) or one-way (A, B, C, H, I) ANOVA comparisons are shown. Data are mean ± s.e.m.; **, *P* ≤ 0.01; ***, *P* ≤ 0.001; ****, *P* ≤ 0.0001; ns, nonsignificant.

Naturally occurring small molecules such as flavonoids and flavonoid-like compounds (e.g., Resveratrol) can modulate GJIC (9). Here we initially posited that pharmacological enhancement of GJIC could be used to increase cGAMP-mediated transactivation of adjacent cells and raise antiviral activity. We initially tested a small panel of flavonoid compounds (Genistein, Apigenin, Quercetin, Resveratrol, Kaempferol, and Epigallocatechin gallate [EGCG]) in a coculture model of GJIC. STING-deficient cGAS-overexpressing human cells (HEK-cGAS^low^ herein), which make constitutive levels of cGAMP, were cultured together with STING competent mouse L929 cells stably expressing an IFN-sensitive responsive element [ISRE] luciferase reporter, referred to as LL171 herein (6, 10). Pretreatment of the HEK-cGAS^low^ cells with the flavonoid compounds potentiated ISRE-luciferase expression, except for Genistein which had an inhibitory effect (Fig. 1A). Conversely, Genistein direct treatment of LL171 cells rather induced ISRE-luciferase expression (Fig. 1B). Although not exhibiting any visible toxicity on the HEK-cGAS^low^ cells at the doses used, Genistein treatment reduced cell proliferation above 30 μM (Fig. S1A). To circumvent this, we normalized the number of cGAMP-producing cells after Genistein treatment and confirmed a dose-dependent inhibition of cGAMP transactivation of cocultured LL171 cells by Genistein (Fig. 1C). Similarly, pretreatment of connexin (CX) expressing human MG-63 cells with Genistein (11), that were subsequently transfected with an immunostimulatory DNA (ISD) to activate cGAS (12), significantly decreased ISRE-luciferase expression from cocultured LL171 cells (Fig. 1D). It is noteworthy that intracellular levels of cGAMP were increased by Genistein in HEK cGAS^low^ cells (Fig. 1E), indicating an inhibition of GJIC aligned with the accumulating cGAMP levels observed. Accordingly, GJIC-dependent transfer of the small molecular weight dye Calcein was decreased when the Calcein-loaded HEK donor cells had previously been treated with Genistein (Fig. 1F). Genistein inhibition of Calcein transfer was seen in HEK-STING cells naturally expressing connexin (CX) 43 and 45 (HEK-STING herein) (6) and was comparable to that obtained with carbenoxolone (CBX), a known inhibitor of connexin-forming gap junctions (6, 7) (Fig. 1F). However, HEK-STING CX43/45 double knockout cells (HEK-STING CX KO herein) failed to show any Calcein transfer, suggesting an effect of Genistein at the level of CX43/45 (Fig. 1F). In agreement with this concept, Genistein treatment of SV40T immortalized mouse embryonic fibroblasts (MEFs) significantly decreased CX43 levels by ~60%, as measured by Western blotting (CX43 was undetectable in HEK cells with this method) (Fig. 1G and Fig. S1B).

**FIG 2:**
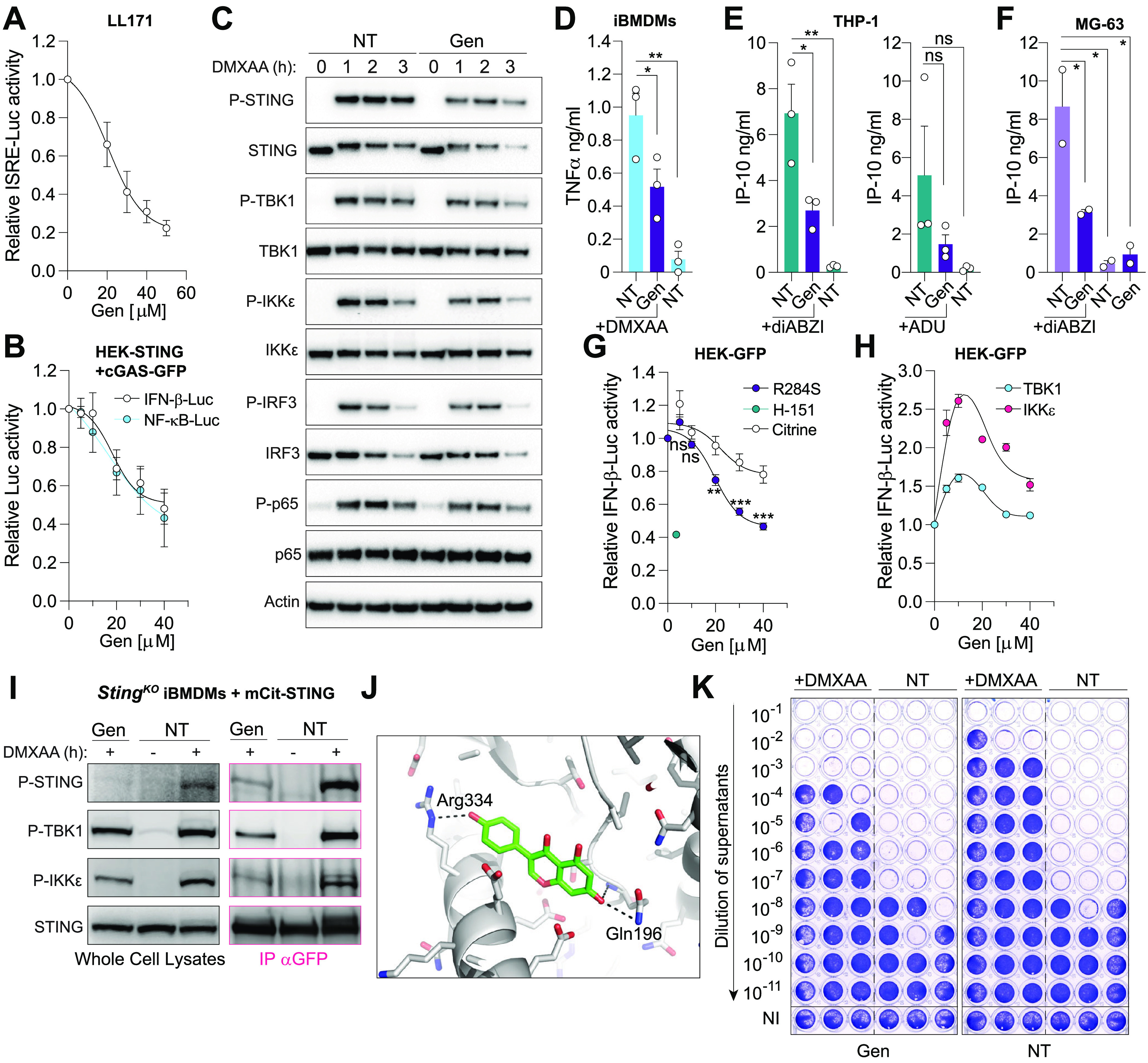
Genistein inhibits STING sensing of its ligands. (A) LL171 cells were treated for 1 h with indicated dose of Genistein followed by DMXAA stimulation (20 μg/mL) for ~8 h. ISRE-luciferase levels were normalized to the “DMXAA only” condition, after background correction with NT condition (*n* = 4). (B) HEK-STING cells transiently overexpressing cGAS-GFP, and an IFN-β-luciferase or an NF-κB-luciferase reporter were treated overnight with indicated dose of Genistein. The data are shown reported to the “NT” condition (*n* = 3 for IFN-β-luciferase and *n* = 2 for NF-κB-luciferase). (C) iBMDMs were pretreated or not for 1 h with 40 μM Genistein, washed and treated with 50 μg/mL DMXAA for indicated times before lysis and immunoblotting (*n* = 3). (D) iBMDM cells were pretreated or not for 1 h with 40 μM Genistein prior to overnight stimulation with 50 μg/mL DMXAA and mouse TNF-α levels measured by specific ELISA (*n* = 3). (E) Undifferentiated THP-1 cells were pretreated or not for 1 h with 40 μM Genistein before 6 h stimulation with 100 nM diABZI (left) or 30 μM ADU-S100 (right), and IP-10 levels were analyzed by specific ELISA (*n* = 3). (F) MG-63 cells were pretreated or not for 1 h with 40 μM Genistein, followed by 7 h stimulation with 100 nM diABZI, and IP-10 levels were analyzed by specific ELISA (*n* = 2). (G, H) HEK293T-GFP (HEK-GFP) transiently expressing an IFN-β-luciferase reporter with either Citrine (control), STING(R284S) (G), TBK1, or IKKε vectors (H), were treated overnight with indicated dose of Genistein or 3.6 μM H-151 (STING inhibitor). Data are shown relative to the “NT” condition (*n* = 3). (I) Immunoblot of *Sting*^KO^ iBMDMs expressing mCitrine-STING, after 1 h stimulation with 50 μg/mL DMXAA and 150 μM Genistein. Pink: pull-down of mCitrine-STING with anti-GFP antibody. Black: whole cell lysates (*n* = 3). (J) *In silico* docking of Genistein with a STING dimer—the model shown is that of pose 1, with the most favorable binding energy (see Text S1 and Fig. S3). Genistein is shown as sticks and 3 h-bonds are indicated as dashed lines. (K) LL171 cells were pretreated for 1 h with or without 40 μM Genistein followed by stimulation with 20 μg/mL DMXAA. After 4 h stimulation with DMXAA, the cells were washed and infected in biological triplicate with Semliki Forest virus (SFV) for 24 h (MOI 2). Viral titers were assayed with log_10_ fold dilutions on confluent Vero cells as shown. NI: not infected (uninfected cells stain with crystal violet); NT: nontreated prior infection. Data shown are representative of three independent experiments. (D, E, F) Each point represents the mean data for each independent experiment. Two-way (G) or one-way (D, E, F) ANOVA comparisons are shown. Data are mean ± s.e.m.; *, *P* ≤ 0.05; **, *P* ≤ 0.01; ***, *P* ≤ 0.001; ns, is nonsignificant.

10.1128/mbio.02064-22.1 FIG S1 (A) HEK-cGAS^low^ cells were treated with indicated amount of Genistein for 48 h, prior to being collected and counted on a hemocytometer. Cell counts were normalized to the NT condition (*n* = 3). (B) Densitometry analyses of CX43 levels from Fig. 1G, in MEFs treated for 48 h with 50 μM Genistein (Gen). The adjusted volume intensities (see Supplemental Methods) of the P0 bands of CX43 (most abundant band with the lowest molecular weight) were normalized to those obtained for tubulin, and further reported to the “NT” condition (*n* = 2). (C) Densitometry analyses of P-STING levels from Fig. 2C, in iBMDMs pre-treated (+) or not (-) for 1 h with 40 μM Genistein, washed and treated with 50 μg/mL DMXAA for indicated times. The adjusted volume intensities of the P-STING bands were normalized to those obtained for actin, and further reported to the “NT 1 h” condition (*n* = 3). (A, B, C) Each point represents one independent experiment. One-way ANOVA comparisons (A) or unpaired two-tailed t-tests (C) are shown. Data are mean ± s.e.m. Symbols used: *, *P* ≤ 0.05 and “ns” is non-significant. Download FIG S1, PDF file, 0.1 MB. Copyright © 2022 Ullah et al. 2022 Ullah et al. https://creativecommons.org/licenses/by/4.0/ This content is distributed under the terms of the Creative Commons Attribution 4.0 International license.

Gap junctions are formed by two connexin-forming hemichannels, with one contributed by each adjacent cell to ensure intercellular communication of cGAMP (6, 10). Surprisingly, Genistein pretreatment of the MEFs entirely ablated the transactivation from the cocultured HEK-cGAS^low^ cells, measured by the secretion of mouse IP-10 (Fig. 1H). This strong inhibitory effect was unexpected given that pretreatment of donor cGAMP-producing HEK-cGAS^low^ cells only partially decreased transactivation of the recipient cells (Fig. 1C). Critically, pretreatment of the MEFs or LL171 cells significantly blunted IP-10 production or ISRE-luciferase expression upon ISD transfection (Fig. 1I and J) suggesting a direct inhibition of STING sensing independent of CX inhibition.

In agreement with this, we observed a dose-dependent inhibitory activity of Genistein on ISRE-Luciferase in LL171 cells stimulated with the murine STING agonist DMXAA (Fig. 2A). To better define how Genistein impacts STING downstream signaling upon activation, we assessed its activity on NF-κB-Luciferase and IFN-β-Luciferase levels in HEK-STING cells that make endogenous cGAMP upon overexpression of cGAS-GFP (7, 13). Genistein inhibited both branches of STING signaling equivalently (Fig. 2B), indicating that its effect was mediated upstream of IRF3 and NF-κB. Western blot analysis of Genistein pretreated immortalized mouse bone marrow-derived macrophages (iBMDMs) stimulated with DMXAA showed a 40 to 50% decrease of STING phosphorylation, and, to a lesser degree, IKKε and TBK1, which resulted in decreased IRF3 (most visible at 1 h) and p65 phosphorylation (most visible at 2 h) (Fig. 2C and Fig. S1C). This was concurrent with a significant decrease of TNF-α production by the iBMDMs (Fig. 2D). Critically, Genistein pretreatment also strongly reduced IP-10 production by human monocytic THP-1 cells stimulated with a cGAMP analog (ADU-S100) or a synthetic STING agonist (diABZI) (14) (Fig. 2E)—the latter observation being replicated in MG-63 cells (Fig. 2F). Genistein also reduced IFN-β-Luciferase driven by overexpression of a gain of function STING variant R284S (1, 15) (Fig. 2G).

10.1128/mbio.02064-22.5 TEXT S1 Experimental details of the reagents and methods used. Download Text S1, PDF file, 0.1 MB. Copyright © 2022 Ullah et al. 2022 Ullah et al. https://creativecommons.org/licenses/by/4.0/ This content is distributed under the terms of the Creative Commons Attribution 4.0 International license.

Mechanistically, Genistein’s inhibitory activity is not targeted at the level of STING trafficking as evidenced by the unaltered formation of STING foci in HEK-STING cells following stimulation with diABZI (Fig. S2A). Similarly, Genistein did not decrease IFN-β-Luciferase driven by overexpression of human TBK1 or IKKε (Fig. 2H). Nonetheless, Genistein decreased the interaction of mCitrine tagged STING with phospho-TBK1 and IKKε following stimulation of iBMDMs with DMXAA and pull-down of mCitrine-STING (Fig. 2I). This suggests that Genistein limits STING signaling through the modulation of the STING/TBK1/IKKε interactions, presumably through its interaction with STING since it did not affect signaling driven by overexpression of human TBK1 or IKKε. Accordingly, *in silico* molecular docking of Genistein with STING identified 3 locations where Genistein was predicted to bind. We obtained 9 binding poses of interaction, and that with the lowest binding energy score, located in proximity to the STING C-terminal tail required for TBK1 recruitment (16), is shown in Fig. 2J and in Fig. S3. This illustrates the potential for a direct interaction of Genistein with STING. Albeit warranting further validation, these results collectively suggest that Genistein impacts STING signaling by direct targeting and preventing its interaction with TBK1/IKKε. It is also noteworthy that beyond its effect on STING signaling, Genistein also decreased IFN-β-Luciferase upon TLR3 activation, presumably due to its inhibitory effect on NF-κB activity (17) (Fig. S2B).

10.1128/mbio.02064-22.2 FIG S2 (A) HEK-STING cells were pretreated with 40 μM Genistein for 1 h, followed by stimulation with 400 nM diABZI STING agonist for 3.5 h. The proportion of cells with STING puncta was then assessed by fluorescent microscopy (*n* = 3). (B) HEK-TLR3 cells transiently expressing an IFN-β-luciferase reporter were pretreated with 40 μM Genistein for 1 h prior to stimulation with 0.5 μg/mL of polyI:C (pIC) for 7 h. Background corrected data are shown relative to the “pIC” condition (*n* = 3). Data are mean ± s.e.m. One-way ANOVA comparisons are shown. Symbol used: ****, *P* ≤ 0.0001. Download FIG S2, PDF file, 0.1 MB. Copyright © 2022 Ullah et al. 2022 Ullah et al. https://creativecommons.org/licenses/by/4.0/ This content is distributed under the terms of the Creative Commons Attribution 4.0 International license.

10.1128/mbio.02064-22.3 FIG S3 Docked poses of Genistein on the surface of a STING dimer (blue and green colours depict individual STING monomers). Models: 1, 2, 3, 4, 7 & 8 show Genistein placed in equivalent positions – circled in blue. Models: 5, & 6 show Genistein in equivalent positions – circled in red. Model 9 was unique (circled in orange). Model 9 places Genistein the closest to ligand binding site of STING at the dimer interface. The energy score for each model is provided (kcal/mol). Download FIG S3, PDF file, 0.6 MB. Copyright © 2022 Ullah et al. 2022 Ullah et al. https://creativecommons.org/licenses/by/4.0/ This content is distributed under the terms of the Creative Commons Attribution 4.0 International license.

Finally, to determine whether the inhibitory effect of Genistein on STING signaling sensitized cells to viral infections, we tested whether it was able to block the antiviral activity of the STING agonist DMXXA. While DMXAA priming of LL171 cells promoted a strong antiviral effect against infection by the RNA Semliki Forest Virus (SFV), pretreatment with Genistein prior to DMXAA reduced this antiviral effect (Fig. 2K and Fig. S4A, S4B). This proviral effect of Genistein was comparable to that obtained with anti-IFNAR1 antibody treatment. Accordingly, the combination of Genistein with the anti-IFNAR1 antibody did not result in a robust increase of viral replication (Fig. S4B). This supports the predominant proviral effects of Genistein seen in this system as relying on type-I IFN inhibition. However, we note that Genistein modestly increased the proviral effect of the anti-IFNAR1 antibody, presumably due to its inhibitory impact on NF-κB activity (17) (Fig. S4B).

10.1128/mbio.02064-22.4 FIG S4 (A) LL171 cells were pre-treated for 1 h with or without 40 μM Genistein, in the presence of 10 μg/mL anti-IFNAR1 antibody (αIFNAR1, clone MAR1-5A3) or isotype control antibody, followed by stimulation with 20 μg/mL DMXAA. After 4 h stimulation with DMXAA, the cells were washed and infected in biological triplicate with Semliki Forest virus (SFV) for 24 h (MOI 2). Viral titers were assayed with log_10_ fold dilutions on confluent Vero cells as shown. NI: not infected (uninfected cells stain with crystal violet); NT: non-treated prior infection. Data shown are representative of three independent experiments. (B) Viral titers from (A) were reported to the non-Genistein +DMXAA treated condition with isotype control antibody treatment. Each independent viral experiment is highlighted with a different color. Download FIG S4, PDF file, 0.4 MB. Copyright © 2022 Ullah et al. 2022 Ullah et al. https://creativecommons.org/licenses/by/4.0/ This content is distributed under the terms of the Creative Commons Attribution 4.0 International license.

Our findings collectively establish the proviral activity of Genistein through its inhibition the cGAS-cGAMP-STING pathway at two levels. On one hand, Genistein can block the transfer of cGAMP to adjacent cells, through the reduction of GJIC. This diminishes the amplification of antiviral responses mediated by cGAMP intercellular transfer (6, 7). Mechanistically, our data in MEFs suggest that this effect is driven by a decrease in CX43 mediated by Genistein. However, cGAMP can also be transferred between cells through hemichannels formed by CX26, CX31, CX32, CX40 and CX62 (6). Therefore, it remains to be determined whether hemichannels beyond those involving CX43 are also targeted by Genistein. On the other hand, Genistein has been shown to directly inhibit STING activation by its agonists (18), which we posit results from a direct interaction of Genistein with STING, thereby impeding its interaction with TBK1 and IKKε. However, further studies will be required to ascertain whether this activity of Genistein also modulates STING-driven autophagy and apoptosis. We also showed that Genistein has a small effect on TLR3-driven antiviral responses suggesting it may have broad immunosuppressive effects on IFN-β production beyond STING. Importantly, Genistein has been shown to have protective effects in several animal models of diseases that have now been linked to STING signaling (19), including acute pancreatitis (20), DMBA-induced cancer (21), nonalcoholic fatty liver and alcoholic liver diseases (22, 23).

Several studies have previously reported an antiviral effect of Genistein against DNA viruses such as HSV-1, cytomegalovirus, or Herpes B Virus, leading to the proposition that its topical administration could be a promising antiviral treatment against these viruses (24). Our findings suggest that its inhibitory effects on type-I IFN responses could confound other antiviral activities in the context of DNA viruses that naturally engage this pathway, prompting caution in its therapeutic development as an antiviral.

### Data availability.

Additional numerical data used to generate published graphs that support the findings of this study are available on request from the corresponding author.
