# Supplementary figures and images for: Genistein Targets STING-Driven Antiviral Responses
Source: mBio. 2022 Aug 4;13(4):e02064-22. doi: 10.1128/mbio.02064-22 (PMC9426420; doi:10.1128/mbio.02064-22)

**A****HEK-cGAS<sup>low</sup>**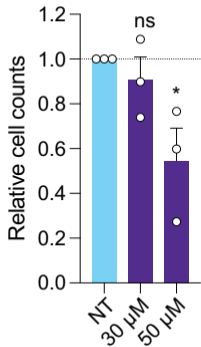**B****MEFs**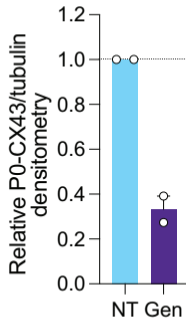**C****iBMDMs**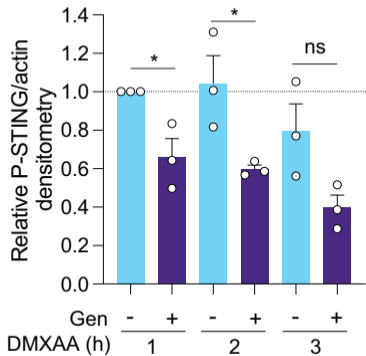**Supplemental Figure S1**

Supplement: FIG S1 [file mbio.02064-22-s0001.pdf]

**A** HEK-STING-mCherry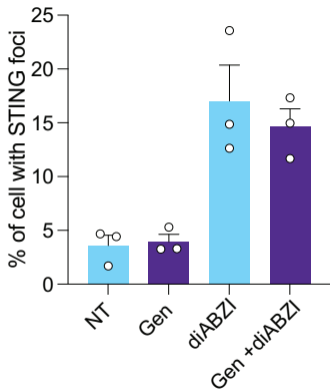**B** HEK-TLR3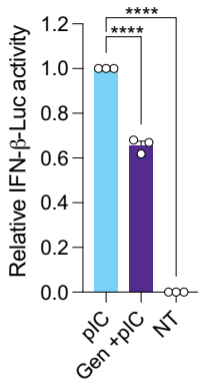**Supplemental Figure S2**

Supplement: FIG S2 [file mbio.02064-22-s0002.pdf]

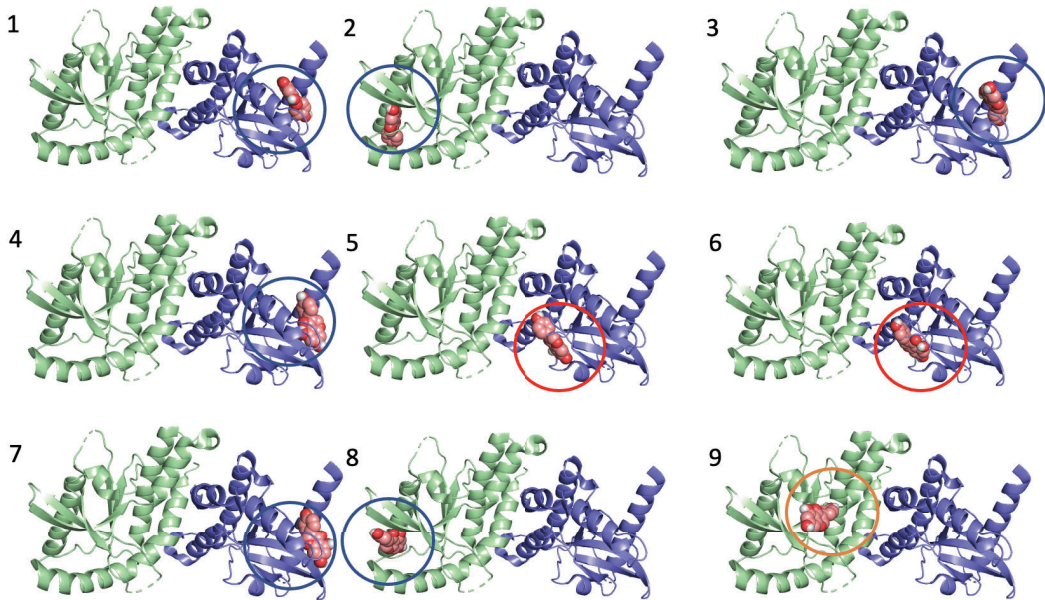

Energy score  
for each model  
(kcal/mol)

1. -7.5

2. -7.4

3. -7.3

4. -7.2

5. -7.2

6. -7.1

7. -7.0

8. -7.0

9. -7.0

**Supplemental Figure S3**

Supplement: FIG S3 [file mbio.02064-22-s0003.pdf]

**A**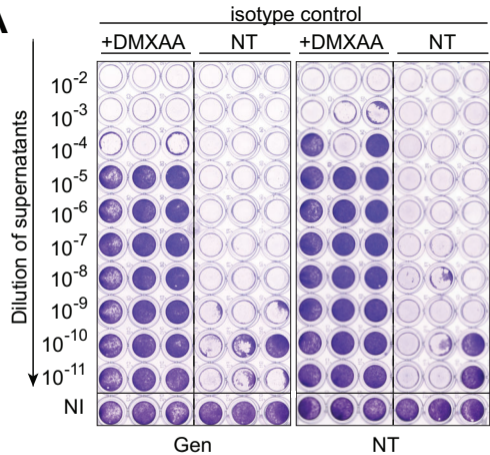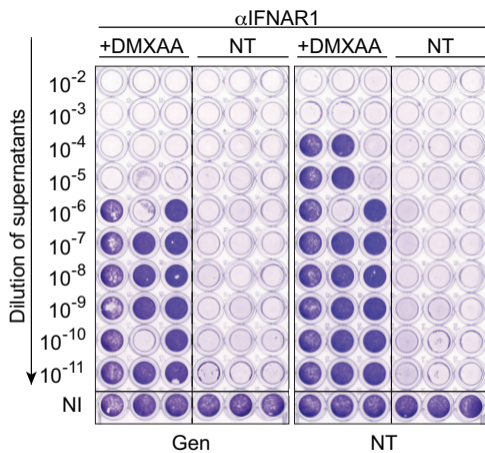**B**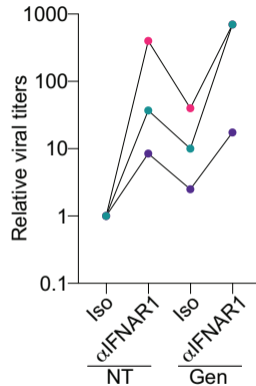**Supplemental Figure S4**

Supplement: FIG S4 [file mbio.02064-22-s0004.pdf]
