# Supplementary material for: Genistein Targets STING-Driven Antiviral Responses
Source: mBio. 2022 Aug 4;13(4):e02064-22. doi: 10.1128/mbio.02064-22 (PMC9426420; doi:10.1128/mbio.02064-22)
Supplement: TEXT S1 [file mbio.02064-22-s0005.pdf]

Supplemental Methods

Cell culture and reagents. HEK-cGAS^{low} cells overexpressing murine cGAS and constitutively producing cGAMP, HEK-STING CX KO cells and matched CX WT (originally reported as HEK-STING CX43/45$^{-/-}$ or CX43/45$^{+/+}$ as they stably express the murine *Sting* fused to an N-terminal mCherry-tag), and LL171 reporter cells (L929 cells expressing an IFN stimulated response element [ISRE]-Luciferase) were all previously described ***(1)***. Wild-type MEFs from C57BL/6 mice immortalized with SV40 large T antigen expression using pSG5-SV40-LT-Ag (gift from D. Huang, Walter and Eliza Hall Institute of Medical Research, Melbourne, Australia) were previously reported ***(2)***. hTLR3 stably expressing human TLR3 were purchased from Invivogen and were maintained in complete DMEM supplemented with 30 µg/ml Blasticidin (Invivogen). J2-retrovirus immortalized bone marrow-derived macrophages from WT C57BL/6 mice ***(3)***, WT MEFs, LL171, Vero cells, Human osteosarcoma MG-63 cells (ATCC #CRL-1427), HEK-cGAS^{low}, HEK293T GFP ***(4)***, HEK-STING CXKO and WT cells were grown in Dulbecco's modified Eagle's medium L-glutamine medium (ThermoFisher Scientific) supplemented with 10 % heat inactivated foetal bovine serum (ThermoFisher Scientific) and 1× antibiotic/antimycotic (ThermoFisher Scientific) (referred to as complete DMEM). THP-1 cells were grown in RPMI 1640 plus L-glutamine medium (ThermoFisher Scientific) complemented with 1x antibiotic/antimycotic and 10% heat inactivated foetal bovine serum (referred to as complete RPMI).

The flavonoid compounds used were from Sigma (all resuspended in DMSO): Genistein (#G6649), Resveratrol (#R5010), Quercetin (#337951), Apigenin (#A3145), Kaemp (#K0133) and EGCG (#E4143). diABZI compound #3 (selleckchem # S8796) and ADU-S100 (MedChemExpress #HY-12885) were used as human STING agonists, and DMXAA (Cayman chemical #14617) as mouse STING agonist. The human STING inhibitor H-151 (Cayman chemical # 25857) and poly(I:C) (Invivogen) were used where indicated.

For GJIC assays, cells were treated with 2 µg/ml Calcein AM solution (Sigma #C1359) and with or without Carbenoxolone (CBX, Sigma #C4790 - resuspended in H_2O). Transfection of IFN stimulatory DNA (ISD) was performed using either a 45 long dsDNA for mouse cell lines (TAC AGA TCT ACT AGT GAT CTA TGA CTG ATC TGT ACA TGA TCT ACA – annealed with complementary sequence) or 70 long dsDNA for human cell lines (CCA TCA GAA AGA GGT TTA ATA TTT TTG TGA GAC CAT CGA AGA GAG AAA GAG ATA AAA CTT TTT TAC GAC T - annealed with complementary sequence) at a concentration of 1-2.5 µg/ml at a ratio of 1 µg:1 µl with Lipofectamine 2000. Intracellular cGAMP was measured by lysing ~200,000 cells in 200 µL of M-PER lysis buffer (ThermoFisher Scientific, #78501) according to the manufacturer's protocol. Cleared lysates (50 µL per sample) were used to quantitate cGAMP using 2'3'-cGAMP ELISA kit (Cayman chemical, #501700).

Luciferase assays

For overexpression of pRP-Citrine (control vector – kind gift from E. Latz, University of Bonn), human STING(R284S) ***(5)***, human TBK1 or human IKKε (in pcDNA3.1 [kind gift from A. Mansell, Hudson Institute]), 400 ng of either vector was co-transfected with 200 ng of reporter (Interferon-β-Luc reporter plasmid [a kind gift from K. Fitzgerald, University of Massachusetts] or pNF-κB-Luc4 reporter [Clontech]), in 750,000 HEK293T-GFP with lipofectamine 2000 in a 6-well plate. The cells were washed 3 h after transfection and plated in 12 wells of a 96 well plate and treated with indicated amount of Genistein or 3.6

μM H-151 overnight. A similar protocol was used for HEK-STING cells co-transfected with murine cGAS-GFP construct ***(6)*** (kind gift from V. Hornung, University of Munich) and IFN-β-Luc. HEK293 cells stably expressing TLR3 were reverse-transfected for 24 h with IFN-β-Luc reporter (Clontech), with Lipofectamine 2000 (Thermo Fisher Scientific), according to the manufacturer's protocol. Following transfection, the cells were collected from the 6-wells and aliquoted into 96-wells, just before overnight poly(I:C) stimulation with or without Genistein. The next day, the cells were lysed in 40 µl (for a 96-well plate) of 1X Glo Lysis buffer (Promega). For LL171 treated alone with DMXAA or following co-culture with HEK-cGAS^{low} or ISD-transfected MG-63 cells, the same protocol was used and the cells were lysed in 40 µl of 1X Glo Lysis buffer. 15 µl of the lysate was then subjected to firefly luciferase assay using 40 µl of Luciferase Assay Reagent (Promega). Luminescence was quantified with a Fluostar OPTIMA (BMG LABTECH) luminometer.

Western blotting. For Figure 1G, MEFs were seeded in 6 well plates at a density of ~500,000 cells per well for 48 h with 50 µM Genistein. Cell lysates were analysed as previously described ***(7)***. Protein detection was carried out using 1:1000 rabbit anti-Connexin 43 (Cell Signalling #3512S) or rat monoclonal anti-tubulin (YL1/2 | ab6160, Abcam). Finally, conjugated secondary antibodies with IRdye800 (Rockland) were used to image the proteins at 800 nm with a ChemicDoc scanner (Bio-Rad). For Figure 2C, 1-1.5 x 10^6 iBMDMs were lysed with 150 µl RIPA buffer supplemented with PMSF, 1x phosSTOP and 1x cOmplete protease inhibitors (Roche). Lysates were cleared at 17,000g for 1 min through Pierce centrifuge columns (ThermoFisher Scientific #89868) before diluting 60 µl with 20 µl of 4x SDS-PAGE sample loading buffer and 10 min denaturation at 95C. Samples were then analysed as previously described ***(8)***. The following antibodies from Cell Signaling were used: STING (#13647S), p-STING (#72971), TBK1 (#3013), p-TBK1 (#5483), IKKε (#3416), p-IKKε (#8766), IRF3 (#4302), p-IRF3 (#4947), p65 (#4764), and p-p65 (#3033); anti-beta ACTIN (Abcam #Ab49900) was used as loading control and Peroxidase-AffiniPure Goat Anti-Rabbit (Jackson ImmunoResearch Labs #111-035-003) was used a secondary antibody used in Chemiluminescent detection (as per ***(8)***). Densitometry analyses were conducted using Image Lab 6.1 software (BioRad) using the "Adj. Volume (Int)" value for each band measured.

Isolation of STING–TBK1 complexes by immunoprecipitation. Immunoprecipitation experiments were performed similarly to those previously described ***(9)***. Approximately 15 × 10^6 *Sting^{KO}* iBMDMs expressing mCitrine-mouse STING were lysed on ice for 30 min with 1 ml of 1× NP-40 buffer (1 % Nonidet-P40, 20 mM Tris-HCl pH 7.4, 150 mM NaCl, 1 mM EGTA, 10 % glycerol, 10 mM NaPPi, 5 mM NaF and 1 mM Na_3VO_4) supplemented with 1 mM PMSF and cOmplete protease inhibitors (Roche Biochemicals). Whole cell lysates were subsequently clarified by centrifugation at 13,000 × *g* for 10 min at 4 °C. Following preparation of samples for immunoblot, 1-2 µg of anti-GFP antibody (Thermo Scientific; clone E36, A-11120) was added to the remaining whole cell lysate. Samples were then incubated at 4 °C for up to 2 h on a rotator before 50 µL of Dynabeads Protein G (Thermo Scientific; 10004D) were added. Samples were then incubated once more at 4 °C for up to 2 h on a rotator before beads were extensively washed with lysis buffer using a DynaMag-2 magnet (Thermo Scientific; 12321D). Proteins were eluted from beads by addition of 35 µL of 2× reducing SDS-PAGE sample loading buffer (2.5 % SDS, 25 % glycerol, 125 mM

Tris-HCl pH 6.8, 0.01 % bromophenol blue, 100 mM dithiothreitol) and heating at 95 °C for 10 min. Samples were then subjected to immunoblotting as described ***(8)***.

Inverted fluorescent microscopy to measure STING foci. HEK-STING cells express a STING-mCherry fusion which can be used to assess STING aggregation upon stimulation ***(10)***. In these experiments, adherent HEK-STING cells were pre-treated or not with Genistein prior to stimulation with 400 nM diABZI for 3.5 h. Three images were taken per condition with an mCherry compatible filter (with >100 cells per image) by inverted fluorescent microscopy, and the number of cells with STING foci was reported to the total number of cells counted for each condition.

Semliki Forest Virus (SFV) infection. 120,000 adherent LL171 cells were pre-treated with or without 40 µM Genistein for 1 h, followed by stimulation with 20 µg/ml DMXAA for 4 h with or without 10 ug/ml of MAR1-5A3 anti-IFNAR antibody or isotype control (both purchased from Leinco). Cells were washed and infected with Semliki Forest virus (SFV) for 2 h in pure DMEM (MOI 2 - as determined by plaque forming units in Vero cells), prior to being washed with 2.5% FCS DMEM and incubated for 22 h. SFV containing supernatants were then assayed for 48 h on confluent Vero cells as previously reported ***(11)***.

Co-culture studies. For Figure 1A, 20,000 HEK-cGAS^{low} cells were pre-treated with indicated concentration of flavonoid compounds for 48 h. At that point the supernatants were removed and 20,000 LL171 cells were added directly on the top of the HEK-cGAS^{low} in each well for overnight incubation. For Figure 1C, HEK-cGAS^{low} were pre-treated with Genistein for 48 h were washed, collected/counted, and 20,000 cells added directly on top of 20,000 adherent LL171 cells plates in a 96 well plate, for overnight incubation. For Figure 1D, MG-63 cells were treated with 30 or 50 µM Genistein for 40 h, washed, collected/counted and 15,000 cells were transfected with IDS70 (2.5 µg/ml) per well of a 96-well plate. 5 h after transfection, the MG-63 cells were washed twice with complete DMEM, and 20,000 LL171 were added directly on the top of the MG-63 cells in each well for overnight incubation. For Figure 1H, MEFs were treated or not with 40 µM Genistein for 40 h, washed, collected/counted and 15,000 cells were added to 20,000 HEK-cGAS^{low} cells in each well for overnight incubation.

Cytokines analysis: Murine IP-10 production in supernatant of MEF cells, co-cultures of MEF cells and HEK-cGAS^{low} was quantified using Mouse CXCL10/IP-10/CRG-2 Duo Set ELISA (R&D systems, #Dy466) according to the manufacturer's protocol. Similarly, human IP-10 production was measured using 100 µL of supernatants from MG-63 or THP-1 cells and were quantified using IP-10 ELISA kit (BD Biosciences, # 555157), according to the manufacturer's protocol. TNF-α levels in culture supernatants from mouse iBMDMs were detected using BD OptEIA Mouse ELISA kit (BD Biosciences) according to the manufacturer's protocol. Tetramethylbenzidine substrate (ThermoFisher Scientific) was used for quantification of the cytokines on a Fluostar OPTIMA (BMG LABTECH) plate-reader.

Molecular docking

The application AutoDockTools ***(12)*** was used to prepare the STING homodimer and ligand to locate potential binding sites of the ligand in the surface of STING (based on charge, hydrophobic interactions and

surface complementarity) using the program Autodock-Vina ***(13)***. The entire surface of STING was explored for ligand binding. The structure of STING was taken from PDB: 4EF4 (waters, calcium ions and its ligand where deleted) and the ligand was downloaded from the Cambridge Crystallographic Data Centre (1165997). The calculation was conducted with rigid side chains of the protein and the ligand was allowed to rotate around its one rotatable bond and hydroxyl groups also allowed to rotate. Nine binding poses were predicted, with varying predicted energies of interactions (see Supplemental Figure S2). None of the predicted sites coincided with the ligand binding site.

Statistical analyses. Statistical analyses were carried out using Prism 9 (GraphPad Software Inc.). All the experiments were conducted at least 3 independent times in biological replicates (unless otherwise stated). One-way and two-way analyses of variance (ANOVA) with multiple comparisons were used when comparing groups of conditions, while unpaired two-tailed t-tests were used when comparing pairs of conditions. Symbols used: * $P \leq 0.05$, ** $P \leq 0.01$, *** $P \leq 0.001$, **** $P \leq 0.0001$ and "ns" is non-significant.

References

1. Ablasser A, Schmid-Burgk JL, Hemmerling I, Horvath GL, Schmidt T, Latz E, Hornung V (2013) Cell intrinsic immunity spreads to bystander cells via the intercellular transfer of cGAMP. Nature 503 (7477):530-534. doi:10.1038/nature12640

2. Pépin G, Nejad C, Thomas BJ, Ferrand J, McArthur K, Bardin PG, Williams BRG, Gantier MP (2017) Activation of cGAS-dependent antiviral responses by DNA intercalating agents. Nucleic Acids Research 45 (1):198-205. doi:10.1093/nar/gkw878

3. Ferrand J, Croft NP, Pépin G, Diener KR, Wu D, Mangan NE, Pedersen J, Behlke MA, Hayball JD, Purcell AW, Ferrero RL, Gantier MP (2018) The Use of CRISPR/Cas9 Gene Editing to Confirm Congenic Contaminations in Host-Pathogen Interaction Studies. Frontiers in Cellular and Infection Microbiology 8. doi:10.3389/fcimb.2018.00087

4. Gantier MP, Tong S, Behlke MA, Irving AT, Lappas M, Nilsson UW, Latz E, McMillan NAJ, Williams BRG (2010) Rational Design of Immunostimulatory siRNAs. Molecular Therapy 18 (4):785-795. doi:10.1038/mt.2010.4

5. Saldanha RG, Balka KR, Davidson S, Wainstein BK, Wong M, Macintosh R, Loo CKC, Weber MA, Kamath V, Moghaddas F, De Nardo D, Gray PE, Masters SL (2018) A Mutation Outside the Dimerization Domain Causing Atypical STING-Associated Vasculopathy With Onset in Infancy. Frontiers in Immunology 9. doi:10.3389/fimmu.2018.01535

6. Pépin G, De Nardo D, Rootes CL, Ullah TR, Al-Asmari SS, Balka KR, Li H-M, Quinn KM, Moghaddas F, Chappaz S, Kile BT, Morand EF, Masters SL, Stewart CR, Williams BRG, Gantier MP, Horner SM, Gale M, Silverman R (2020) Connexin-Dependent Transfer of cGAMP to Phagocytes Modulates Antiviral Responses. mBio 11 (1). doi:10.1128/mBio.03187-19

7. Pepin G, Ferrand J, Honing K, Jayasekara WS, Cain JE, Behlke MA, Gough DJ, Williams BRG, Hornung V, Gantier MP (2016) Cre-dependent DNA recombination activates a STING-dependent innate immune response. Nucleic Acids Res 44 (11):5356-5364. doi:10.1093/nar/gkw405

8. Balka KR, Louis C, Saunders TL, Smith AM, Calleja DJ, D'Silva DB, Moghaddas F, Tailler M, Lawlor KE, Zhan Y, Burns CJ, Wicks IP, Miner JJ, Kile BT, Masters SL, De Nardo D (2020) TBK1 and IKKε Act Redundantly to Mediate STING-Induced NF-κB Responses in Myeloid Cells. Cell Reports 31 (1):107492. doi:10.1016/j.celrep.2020.03.056

9. De Nardo D, Balka KR, Cardona Gloria Y, Rao VR, Latz E, Masters SL (2018) Interleukin-1 receptor–associated kinase 4 (IRAK4) plays a dual role in myddosome formation and Toll-like receptor signaling. Journal of Biological Chemistry 293 (39):15195-15207. doi:10.1074/jbc.RA118.003314

10. Ablasser A, Schmid-Burgk JL, Hemmerling I, Horvath GL, Schmidt T, Latz E, Hornung V (2013) Cell intrinsic immunity spreads to bystander cells via the intercellular transfer of cGAMP. Nature 503 (7477):530-534. doi:10.1038/nature12640
11. Pépin G, Ferrand J, Höning K, Jayasekara WSamantha N, Cain JE, Behlke MA, Gough DJ, G. Williams BR, Hornung V, Gantier MP (2016) Cre-dependent DNA recombination activates a STING-dependent innate immune response. Nucleic Acids Research 44 (11):5356-5364. doi:10.1093/nar/gkw405
12. Morris GM, Huey R, Lindstrom W, Sanner MF, Belew RK, Goodsell DS, Olson AJ (2009) AutoDock4 and AutoDockTools4: Automated docking with selective receptor flexibility. Journal of Computational Chemistry 30 (16):2785-2791. doi:10.1002/jcc.21256
13. Trott O, Olson AJ (2009) AutoDock Vina: Improving the speed and accuracy of docking with a new scoring function, efficient optimization, and multithreading. Journal of Computational Chemistry:NA-NA. doi:10.1002/jcc.21334
